# Protease inhibitor monotherapy for long-term management of HIV infection: a randomised, controlled, open-label, non-inferiority trial

**DOI:** 10.1016/S2352-3018(15)00176-9

**Published:** 2015-09-14

**Authors:** Nicholas I Paton, Wolfgang Stöhr, Alejandro Arenas-Pinto, Martin Fisher, Ian Williams, Margaret Johnson, Chloe Orkin, Fabian Chen, Vincent Lee, Alan Winston, Mark Gompels, Julie Fox, Karen Scott, David T Dunn

**Affiliations:** a Medical Research Council Clinical Trials Unit at University College London, London, UK; b Department of Medicine, Yong Loo Lin School of Medicine, National University of Singapore, Singapore; c Brighton and Sussex University Hospitals NHS Trust, Brighton, UK; d Research Department of Infection and Population Health, University College London, London, UK; e Royal Free Hospital, London, UK; f Barts and the Royal London Hospital NHS Trust, London, UK; g Royal Berkshire Hospital, Reading, UK; h Manchester Royal Infirmary, Manchester, UK; i St Mary's Hospital, London, UK; j Southmead Hospital, Bristol, UK; k Guy's and St Thomas' Hospital, London, UK

## Abstract

**Background:**

Standard-of-care antiretroviral therapy (ART) uses a combination of drugs deemed essential to minimise treatment failure and drug resistance. Protease inhibitors are potent, with a high genetic barrier to resistance, and have potential use as monotherapy after viral load suppression is achieved with combination treatment. We aimed to assess clinical risks and benefits of protease inhibitor monotherapy in long-term clinical use: in particular, the effect on drug resistance and future treatment options.

**Methods:**

In this pragmatic, parallel-group, randomised, controlled, open-label, non-inferiority trial, we enrolled adults (≥18 years of age) positive for HIV attending 43 public sector treatment centres in the UK who had suppressed viral load (<50 copies per mL) for at least 24 weeks on combination ART with no change in the previous 12 weeks and a CD4 count of more than 100 cells per μL. Participants were randomly allocated (1:1) to maintain ongoing triple therapy (OT) or to switch to a strategy of physician-selected ritonavir-boosted protease inhibitor monotherapy (PI-mono); we recommended ritonavir (100 mg)-boosted darunavir (800 mg) once daily or ritonavir (100 mg)-boosted lopinavir (400 mg) twice daily, with prompt return to combination treatment if viral load rebounded. All treatments were oral. Randomisation was with permuted blocks of varying size and stratified by centre and baseline ART; we used a computer-generated, sequentially numbered randomisation list. The primary outcome was loss of future drug options, defined as new intermediate-level or high-level resistance to one or more drugs to which the patient's virus was deemed sensitive at trial entry (assessed at 3 years; non-inferiority margin of 10%). We estimated probability of rebound and resistance with Kaplan-Meier analysis. Analyses were by intention to treat. This trial is registered with the International Standard Randomised Controlled Trial Number registry, number ISRCTN04857074.

**Findings:**

Between Nov 4, 2008, and July 28, 2010, we randomly allocated 587 participants to OT (291) or PI-mono (296). At 3 years, one or more future drug options had been lost in two participants (Kaplan-Meier estimate 0·7%) in the OT group and six (2·1%) in the PI-mono group: difference 1·4% (−0·4 to 3·4); non-inferiority shown. 49 (16·8%) participants in the OT group and 65 (22·0%) in the PI-mono group had grade 3 or 4 clinical adverse events (difference 5·1% [95% CI −1·3 to 11·5]; p=0·12); 45 (six treatment related) and 56 (three treatment related) had serious adverse events.

**Interpretation:**

Protease inhibitor monotherapy, with regular viral load monitoring and prompt reintroduction of combination treatment for rebound, preserved future treatment options and did not change overall clinical outcomes or frequency of toxic effects. Protease inhibitor monotherapy is an acceptable alternative for long-term clinical management of HIV infection.

**Funding:**

National Institute for Health Research.

## Introduction

Current HIV treatment guidelines recommend a combination of two drug classes for initiation and maintenance of antiretroviral therapy (ART).^1^, ^2^ The principle of combining drugs with different mechanisms of action to increase potency and reduce selection of drug-resistant mutants is common to the treatment of many infectious diseases. However, in HIV, the need for combination treatment might decrease once viral load has been suppressed.

Protease inhibitors are potent, with a high genetic barrier to resistance, and are the only drugs that act at many steps of the HIV lifecycle, thus having potential for use alone as monotherapy.^3^ Protease inhibitor monotherapy could be an attractive therapeutic option because of its potential to reduce renal, CNS, and other toxic effects associated with drugs widely used in standard ART combinations. The high genetic barrier to resistance might reduce the risk of resistance during periods of suboptimum treatment adherence. Furthermore, use of a single drug might decrease treatment costs. Although inadequate for initial treatment,^4^ findings from previous randomised trials of maintenance protease inhibitor monotherapy^5^, ^6^, ^7^, ^8^, ^9^ have shown high levels of short-term viral load suppression. However, these trials have not been of sufficient size and duration to address definitively the effects on long-term drug resistance, clinical disease progression, and drug toxic effects in clinical practice.^5^, ^6^, ^7^, ^8^, ^9^ Furthermore, investigators have mostly restricted the standard-of-care treatment to a specific protease inhibitor regimen and mandated use of a particular protease inhibitor for monotherapy, neither of which takes account of the diversity of regimen selection in routine clinical practice.

**Research in context**

**Evidence before this study**

We searched PubMed for reports published between Jan 1, 1998, and Jan 1, 2008, with no language restrictions, using terms including “protease inhibitors”, “monotherapy”, and the individual drug names, and reviewed relevant HIV conference abstracts to identify randomised controlled trials that compared protease inhibitor monotherapy with triple antiretroviral therapy (ART) in patients who had previously achieved viral load suppression. We identified three trials, all investigating lopinavir monotherapy, and, overall, these trials supported the hypothesis of virological non-inferiority of monotherapy to triple therapy. Since then, authors of a meta-analysis of ten trials noted an overall risk ratio of 0·94 for viral load suppression at 48 weeks with protease inhibitor monotherapy compared with triple ART, with, at most, a 13% increase in the absolute risk of virological failure with protease inhibitor monotherapy.

**Added value of this study**

To our knowledge, this trial is the largest and longest-duration protease inhibitor monotherapy trial done so far, with more than three times the randomly allocated person follow-up time of previous trials, and thus it provides more precise estimates of important but uncommon efficacy and safety outcomes than did previous trials. By contrast with previous studies that restricted the choice of drugs in both the monotherapy (single specified protease inhibitor) and combination ART (usually to a protease-inhibitor-containing combination, and with the same protease inhibitor as specified for monotherapy) groups, this trial used a pragmatic design representative of routine clinical care, with flexibility of drug selection in both treatment groups. Also, whereas previous trials compared predefined regimens with short-term viral load endpoints, this trial compared treatment strategies with a primary efficacy endpoint (preservation of drug options) relevant to long-term clinical management. In this trial we noted an increase of about 32% in absolute risk of virological failure with protease inhibitor monotherapy (much greater than that previously reported), but all patients with rebound resuppressed spontaneously or with reintroduction of combination ART. Findings from this trial showed that the protease inhibitor monotherapy strategy was non-inferior to triple ART in preservation of future treatment options and did not change overall clinical outcomes or frequency of toxic effects.

**Implications of all the available evidence**

Protease inhibitor monotherapy does not increase the risk of drug resistance and is an acceptable alternative for long-term clinical management of HIV infection. Clinical benefits, if any, seem slight, and some patients might need to switch back to combination ART. Nevertheless, this approach could appeal to patients who are stable on treatment but who wish to minimise their exposure to specific drugs or more than one drug class.

We did a pragmatic randomised controlled trial comparing a protease inhibitor monotherapy treatment strategy with clinician-selected standard combination treatment, aiming to assess effects on drug resistance, future treatment options, and other, long-term, clinically relevant outcomes.

## Methods

### Study design and patients

In this pragmatic, parallel-group, randomised, controlled, open-label, non-inferiority trial, we enrolled participants attending 43 public sector hospital-based HIV treatment centres in the UK. Eligible participants were adults aged 18 years or older who were HIV positive, had been on ART consisting of two nucleoside reverse transcriptase inhibitors (NRTIs) and one non-NRTI (NNRTI) or protease inhibitor for at least 24 weeks with no change in the previous 12 weeks, had a viral load of less than 50 copies per mL at screening and for at least 24 weeks before screening (one blip to less than 200 copies per mL allowed during this period if followed by at least two results less than 50 copies per mL), had a CD4 count of more than 100 cells per μL at screening, and who were willing to continue current ART or change according to the randomised allocation. Exclusion criteria were known major protease inhibitor resistance mutations at previous resistance testing (if done; not mandated), previous ART change for unsatisfactory virological response (ie, slow initial virological suppression, incomplete suppression, or rebound; change for convenience or toxic effect prevention or management allowed), protease inhibitor allergy, concomitant drugs with protease inhibitor interactions, present or anticipated need for radiotherapy or cytotoxic chemotherapy, treatment for acute opportunistic infection within the previous 3 months, present or planned pregnancy, active substance misuse or psychiatric illness, history of HIV encephalopathy with a present deficit of more than 1 in any domain of the Neuropsychiatric AIDS Rating Scale,^10^, ^11^ history of cardiovascular disease or a 10 year absolute coronary heart disease risk of more than 30% or more than 20% with diabetes or a family history of premature ischaemic heart disease or stroke,^12^ insulin-dependent diabetes mellitus, active or planned hepatitis C virus treatment, hepatitis B surface antigen positive at screening or since HIV diagnosis (unless hepatitis B DNA of less than 1000 copies per mL taken while off drugs active against hepatitis B), or a fasting plasma glucose of more than 7·0 mmol/L at screening.

The protocol was approved by the Cambridgeshire 4 Research Ethics Committee and Medicines and Healthcare Products Regulatory Agency. All participants provided written informed consent.

### Randomisation and masking

Participants were randomly assigned (1:1) to maintain ongoing triple therapy (OT) or switch to a protease inhibitor monotherapy strategy (PI-mono). Randomisation was stratified by centre (nine groups, based on the eight largest sites and one group for the remaining sites) and baseline ART regimen (protease inhibitor or NNRTI). The computer-generated, sequentially numbered randomisation list (random permuted blocks of varying size) was preprepared by WS. Sites faxed screening forms to the trial coordinating centre, where trial staff confirmed eligibility and did the randomisation (they could access the next number on the list, but not the whole list). Treatment allocation was open label.

### Procedures

In the OT group, we managed patients using triple combination treatment. In the PI-mono group, we switched patients to a single ritonavir-boosted protease inhibitor selected by the physician: we recommended darunavir (800 mg) boosted with ritonavir (100 mg) once daily or lopinavir (400 mg) boosted with ritonavir (100 mg) twice daily. Drugs were taken orally. Patients switching from NNRTI-based regimens continued NRTIs for the first 2 weeks. Protease inhibitor substitution was allowed in the event of toxic effects or for convenience. The strategy required prompt reintroduction of NRTIs (switch of protease inhibitor to NNRTI optional) for protocol-defined confirmed viral load rebound and management with combination treatment for the remainder of the trial (subsequent switches for toxic effects, convenience, and viral load failure allowed, as in OT group).

Study visits at baseline, weeks 4 and 8 (PI-mono group only), week 12, and every 12 weeks thereafter included assessment of clinical status, drug adherence (standardised questions), viral load, CD4 cell count, and safety blood tests (measured at site laboratory). Visits at baseline, week 12, week 48, and every 48 weeks thereafter included additional assessments of cardiovascular disease risk (Framingham equation),^13^ neurocognitive function (standard five-test battery),^14^, ^15^ symptomatic peripheral neuropathy (Brief Peripheral Neuropathy Screen),^16^ and quality of life (self-completed Medical Outcomes Study HIV Health Survey questionnaire^17^, ^18^). We classified clinical and laboratory events with protocol-defined diagnostic criteria (on the basis of Centers for Disease Control and Prevention criteria for AIDS,^19^ INSIGHT criteria for serious non-AIDS events,^20^ and Division of AIDS criteria for adverse events^21^), and an independent physician at the coordinating centre reviewed them. We calculated estimated glomerular filtration rate (eGFR) with the Chronic Kidney Disease Epidemiology Collaboration equation.^22^

If viral load was detectable at 50 copies per mL or higher at any visit, we repeated the test (on the same sample if available or a fresh sample draw if not). If viral load was less than 50 copies per mL on the repeat test, we continued routine follow-up, but if higher, we did adherence counselling and patients returned for a confirmatory test at least 4 weeks from the date that the first sample was taken. If viral load was less than 50 copies per mL with the confirmatory test, we resumed routine follow-up, but if higher (ie, on the third consecutive test), this result met the protocol definition of confirmed rebound and so the patient was required to change treatment, and we did a repeat test 4 weeks later.

We did genotypic resistance testing on all viral load rebound samples that were confirmed or preceded treatment switch. We did genotypic testing at site laboratories, repeated at the central laboratory if local sequencing was unsuccessful. We used the Stanford algorithm for drug susceptibility prediction. If we identified resistance mutations, we compared them with any genotypic testing reports from before the trial.

### Outcomes

The primary outcome was loss of future drug options, defined as new intermediate-level or high-level resistance to one or more drugs in contemporary use to which we deemed patient's virus to be sensitive at trial entry (assessed at 3 years). We defined contemporary use on the basis of inclusion in present UK treatment guidelines,^1^ with saquinavir added because this drug was taken by some participants during the trial.

Secondary outcomes were occurrence of serious drug-related or disease-related complications, including death or serious AIDS-defining (excluding oesophageal candidiasis or chronic mucocutaneous herpes simplex virus infection) or non-AIDS-defining illness; total number of grade 3 and 4 adverse events; confirmed virological rebound; CD4 cell count change from baseline; neurocognitive function change from baseline; cardiovascular risk change from baseline; and health-related quality of life change from baseline (mental and physical health summary scores). Additional specified safety outcomes were facial lipoatrophy, abdominal fat accumulation, peripheral neuropathy, and estimated glomerular filtration rate.

### Statistical analysis

We defined non-inferiority of the PI-mono group by the upper limit of the two-sided 95% CI for the difference in proportions of patients who maintain all future drug options during 3 years (OT group minus the PI-mono group) being less than 10%. We chose the 10% non-inferiority margin on the basis of US Food and Drug Administration guidance 23, 24 and with reference to other protease inhibitor monotherapy trials.^9^ Using a survival analysis approach (on a hazard ratio scale) to allow inclusion of all follow-up data for estimation of the primary endpoint, and assuming that 97% of patients in the OT group would meet the primary endpoint,^7^, ^25^, ^26^ with an 85% power and a 10% loss to follow-up, we estimated that about 280 patients per group would be needed to show non-inferiority. This approach gives a conservative estimate of the sample size, which has the additional advantage of allowing precise estimates of important secondary safety outcomes. On the basis of a conventional analysis of proportions, the power of the study to show non-inferiority for the primary endpoint with use of the same parameters would be more than 99%.

All comparisons were as randomised (intention to treat). We deemed a per-protocol analysis not relevant for this pragmatic trial in view of the fact that a switch back to combination treatment in the PI-mono group was an intrinsic element of the clinical strategy being assessed. Statistical tests are two-sided and test the hypotheses of no difference between randomised groups. We estimated the absolute difference between groups in reduction of future drug options using Kaplan-Meier analysis, with the 95% CI (two-sided) derived with bootstrap methods. In this analysis and other time-to-event analyses, we censored participants at the time of death, loss to follow-up, or withdrawal. The primary analysis included all new resistance mutations noted that conferred intermediate-level or high-level drug resistance. We predefined a sensitivity analysis in which we restricted loss of drug options to classes to which the patient was exposed during the trial, thus excluding mutations that were probably archived (ie, acquired at transmission or during treatment before enrolment, but unknown at trial entry).

For secondary endpoints, we compared binary outcome variables between groups using χ^2^ or Fisher's exact tests, with conventional or Agresti-Caffo 95% CIs for the risk difference and logistic regression models for adjusted analyses. For continuous variables, we compared groups by use of mean change from baseline and *t* tests or linear regression; we estimated change from baseline to the last available visit at which a measurement was done at or after week 144 (we did not include patients without such data). We estimated the proportion of patients who had viral load rebound with Kaplan-Meier analysis and compared groups by use of a log-rank test. We compared incidence rates with Poisson regression. We compared adherence during the entire follow-up period between groups with generalised estimating equations (independent correlation structure; binomial distribution).

We used Stata version 12.1 for all analyses. A data monitoring committee reviewed interim data annually. This trial is registered with the International Standard Randomised Controlled Trial Number registry, number ISRCTN04857074.

### Role of the funding source

The funder of the study had no role in study design, data collection, data analysis, data interpretation, or writing of the report. The corresponding author had full access to all the data in the study and had final responsibility for the decision to submit for publication.

## Results

**Figure 1:**
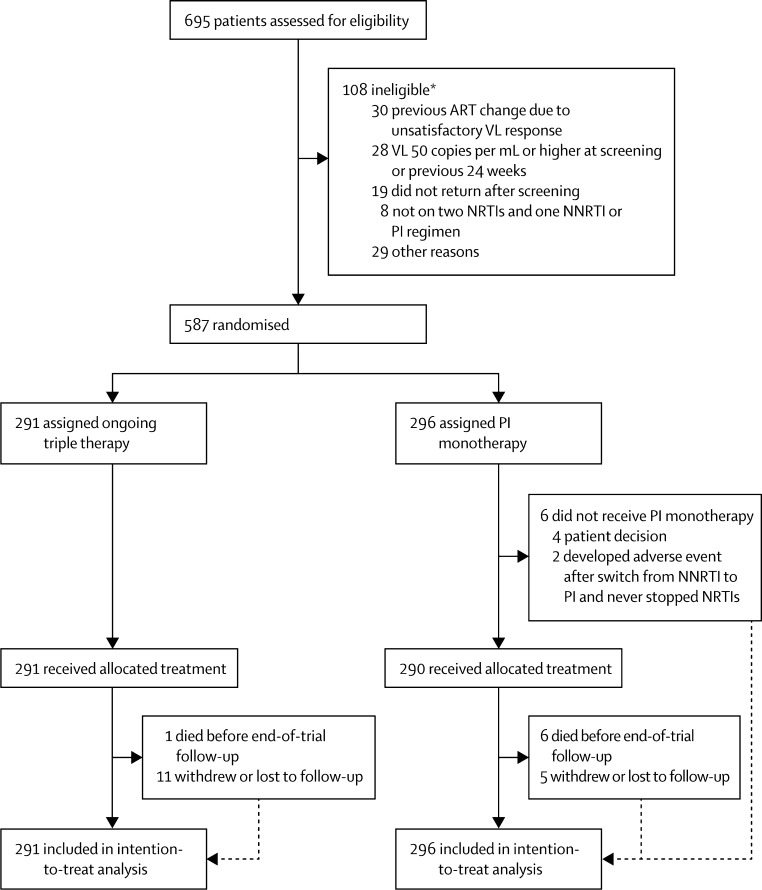
Trial profile ART=antiretroviral therapy. PI=protease inhibitor. NNRTI=non-nucleoside reverse transcriptase inhibitor. NRTI=nucleoside reverse transcriptase inhibitor. VL=viral load. *Six patients had more than one reason for ineligibility.

Between Nov 4, 2008, and July 28, 2010, we randomly allocated 587 participants to OT (291) or PI-mono (296; figure 1). One (<1%) died and 11 (4%) withdrew or were lost to follow-up in the OT group, whereas six (2%) died and five (2%) withdrew or were lost to follow-up in the PI-mono group. Study visits ended on Nov 1, 2013. The median duration of trial follow-up was 44 months (maximum 59 months). Participant characteristics were similar between groups (table 1).

In the PI-mono group, initial drug choices were darunavir in 233 (80%) of patients, lopinavir in 40 (14%), atazanavir in 16 (6%), and saquinavir in 1 (<1%); 58% were still taking monotherapy at trial end. Combination treatment was reintroduced for the following reasons: 69 (23%) for protocol-defined confirmed viral rebound (appendix p 8), 11 (4%) for viral rebound not meeting protocol criteria, 16 (5%) for toxic effects, and 22 (7%) for other or unknown reasons; 6 (2%) never started monotherapy. Overall, 72% of follow-up time was spent on monotherapy. Self-reported adherence to study drugs was high: participants reported not missing any ART doses in the past 2 weeks at 4301 (93%) of 4635 visits in the OT group and 4376 (92%) of 4748 visits in the PI-mono group (p=0·52).

The number of patients with loss of future drug options at 3 years (the primary outcome) was two patients (Kaplan-Meier estimate 0·7%) in the OT group and six (2·1%) in the PI-mono group (difference 1·4% [95% CI −0·4 to 3·4]), therefore meeting the non-inferiority criterion. PI-mono was also non-inferior with prespecified analyses of loss of future drug options during the full trial follow-up period (OT 1·8%; PI-mono 2·1%; difference 0·2% [–2·5% to 2·6]) and loss of future drug options during the full trial period but excluding mutations that were probably archived (OT 1·5%; PI-mono 1·0%; difference −0·4% [–2·9% to 1·4%]). One (<1%) participant on atazanavir monotherapy developed the Ile50Leu mutation (as a mixture with wild-type), conferring predicted high-level atazanavir resistance. We detected an isolated Leu90Met mutation in two (<1%) patients on darunavir monotherapy; both resuppressed with reintroduction of NRTIs. This mutation, possibly archived, does not affect darunavir sensitivity, but confers resistance to saquinavir and thus meets the endpoint definition. Three (1%) patients in the PI-mono group had NRTI or NNRTI mutations detected, probably archived from previous treatment. In the OT group, three (1%) patients had loss of future drug options to drug classes that they were taking and one (<1%) taking a protease-inhibitor-based regimen had NNRTI mutations that were probably archived (table 2; appendix p 2–3).

**Figure 2:**
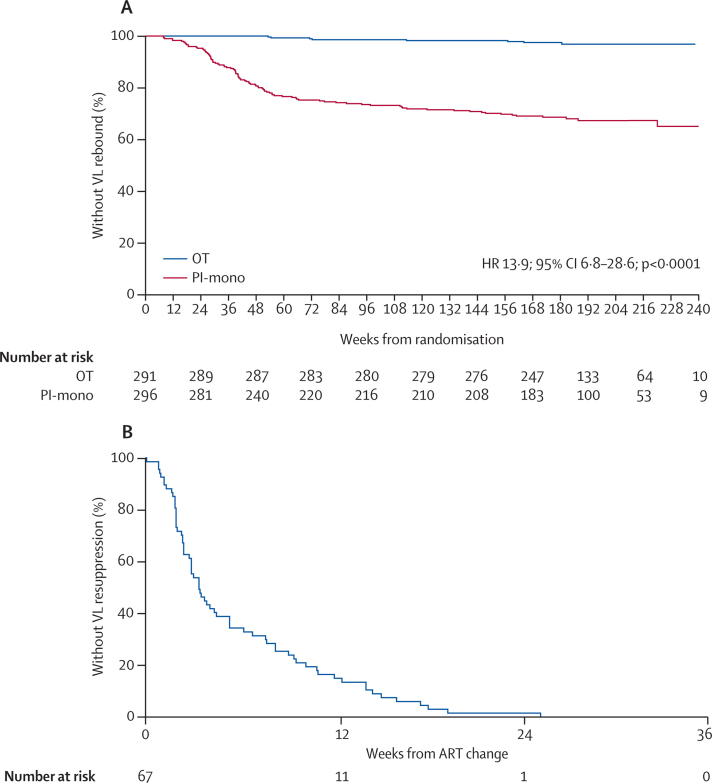
Viral load rebound and resuppression (A) Time to viral rebound. (B) Time to viral resuppression after change of ART in the PI-mono group. The time to first viral load of less than 50 copies per mL (midpoint between last test 50 copies per mL or more and first test of less than 50 copies per mL) for patients at the time of first rebound who were taking protease inhibitor monotherapy (excluding two patients in the PI-mono group who rebounded while not on monotherapy) is shown. Outcomes by type of treatment switch are shown in the appendix (p 8). ART=antiretroviral therapy. HR=hazard ratio. OT=ongoing triple therapy. PI-mono=protease inhibitor monotherapy. VL=viral load.

We noted one or more episodes of confirmed viral load rebound in eight (Kaplan-Meier estimate 3·2%) patients in the OT group and 95 (35·0%) in the PI-mono group during all follow-up: absolute risk difference 31·8% (95% CI 24·6–39·0; p<0·0001). Rebound while actually on monotherapy (occurred in 93 patients) was more common in the first year (24 per 100 person-years) than in subsequent years (six per 100 person-years; figure 2). Findings were similar with use of an extended definition of viral load rebound (appendix, p 9) and across the different protease inhibitors used as monotherapy. The median peak viral load at first episode of rebound on monotherapy was 526 copies per mL (peak was less than 400 copies per mL in 39 [42%] of 93 patients); of the 91 (98%) patients with subsequent tests available, 22 (24%) had resuppressed spontaneously and 69 (76%) resuppressed after changing ART: 47 (68%) by adding NRTIs only, 18 (26%) by adding NRTIs and changing the protease inhibitor to an NNRTI, two (3%) by changing the protease inhibitor monotherapy drug, and two (3%) other changes (appendix p 8). Viral load was resuppressed to less than 50 copies per mL after a median of 3·5 weeks (figure 2).

We noted no differences in serious drug-related or disease-related complications (death, AIDS, or serious non-AIDS) between groups (table 3). Causes of the one death in the OT group and six in the PI-mono group were diverse, and we did not deem any to be related to treatment strategy (appendix p 4). CD4 cell count increased similarly in both groups (table 3). Serious adverse events and clinical grade 3 and 4 adverse events did not differ between groups (table 3, appendix p 5–7). Fewer patients in the PI-mono group had a serum phosphate concentration of less than 0·65 mmol/L (p<0·0001, appendix p 6) and an eGFR of less than 60 mL/min per 1·73 m^2^ during follow-up (table 3). However, mean change in eGFR did not differ between groups (table 3), and only one case of end-stage renal failure occurred: a patient in the PI-mono group with pre-existing chronic renal impairment. Proportions of patients with symptomatic peripheral neuropathy, facial lipoatrophy, or abdominal fat accumulation during follow-up, and changes in neurocognitive function summary, cardiovascular disease risk, or quality of life summary scores did not differ between groups (table 3).

## Discussion

In patients who have achieved viral load suppression with combination treatment, a maintenance strategy of protease inhibitor monotherapy, with reintroduction of combination treatment in the event of viral load rebound, was non-inferior to continuous combination treatment for preservation of future treatment options during 3–5 years. Furthermore, and perhaps more relevant than is non-inferiority for understanding of the clinical implications of this approach, the findings showed that the absolute number of patients who lost future drug options with protease inhibitor monotherapy was very low. Only one patient developed clinically significant resistance to a protease inhibitor taken as monotherapy, an Ile50Leu mutation with atazanavir monotherapy; clinical negative effects are mitigated by an increase in sensitivity to other protease inhibitors conferred by this mutation.^27^ None of the 277 patients taking darunavir or lopinavir (the recommended options for monotherapy in this trial, taken by almost all patients) developed resistance affecting their efficacy. The very low amount of resistance concurs with previous trials of monotherapy (and trials with protease-inhibitor-based combinations),^5^, ^6^, ^7^, ^8^, ^9^ but extend findings from these trials to provide the crucial long-term randomised outcome data from a large pragmatic trial needed to provide confidence in use of protease inhibitor monotherapy in clinical practice.

We sought resistance fastidiously, testing confirmed viral rebound samples irrespective of level of viraemia with standard population sequence genotypic testing. Although protease inhibitor resistance could occur in other regions of the virus, such as *gag* or *env* genes, or in minority species that are not detected by population assays,^3^ previous minority species sequencing in patients with viral rebound on monotherapy has shown only a small excess of resistance compared with that detected by population sequencing.^28^, ^29^ Furthermore, our finding that patients with viral load rebound in the PI-mono group, without exception, achieved full viral load suppression when switched to combination treatment (usually by reintroduction of NRTIs) provides an important assurance that any mutations that were not detected by population sequencing did not affect subsequent treatment efficacy. The follow-up period of 3–5 years, long for an HIV treatment trial, is sufficient to have confidence that preservation of treatment options is likely to be durable in the long term.

A higher proportion of patients in the PI-mono group had viral rebound, as expected, although the difference was much greater than the 10–13% noted in a previous systematic review.^9^ Viral suppression is the traditional outcome variable for comparison of treatment regimens in clinical trials, but is less informative in long-term strategy trials such as this one, especially in view of the different effect of viral rebound on risk of drug resistance for different drug classes. Aside from risk of resistance (shown to be negligible in this study), these brief episodes of low-level viraemia, rapidly reversed by reintroduction of combination treatment, are unlikely to have adverse long-term clinical effects: much higher levels of viraemia are needed than those noted in this study to drive HIV disease progression,^30^ and CD4 cell count increases and total HIV disease-related clinical events were similar between the two groups. Risk of transmission to others arising from such short episodes of low-level viraemia is also likely to be negligible in view of the fact that transmission is exceedingly rare in patients with a low viral load, whether on or off ART.^31^, ^32^ Although more deaths occurred in the PI-mono group, the difference was not statistically significant and the causes were diverse, without a plausible link to the drugs taken or HIV disease progression. This finding is probably a chance imbalance, and follow-up of the cohort continues. Further analyses of predictive factors are planned that might enable identification of patients at low risk of rebound. However, selection factors are unlikely to be able to remove the excess risk entirely, and rebound and resuppression should be viewed as an integral part of the strategy for some patients. After the first year of suppression on monotherapy, the risk of rebound is much reduced, and this factor might increase acceptability for patients.

We noted fewer episodes of renal impairment in the PI-mono group as expected in view of the well-recognised renal toxic effects of NRTIs (especially tenofovir). However, the risk of serious drug toxic effects can be minimised by close laboratory monitoring and pre-emptive treatment changes, and no patients in the OT group developed end-stage renal disease.^33^ This pragmatic trial in which clinicians were allowed to switch drugs at clinical discretion (rather than being restricted to single-protocol-mandated regimens) provides a realistic estimate of the effect of protease inhibitor monotherapy in routine clinical practice, suggesting that benefits in terms of renal toxic effects would be small. We did not find any other clinical advantages of the monotherapy strategy. Treatment costs might be reduced, partly offset by increased monitoring costs, but considerations are complex, and detailed cost-effectiveness analyses will be reported in a separate paper.

A concern with protease inhibitor monotherapy is that suboptimum drug penetration into the CNS might lead to harm.^34^, ^35^ However, we noted no difference in neurocognitive function between groups, nor any significant excess of neurological events in the PI-mono group. We used a small battery of neurocognitive tests (for feasibility in view of the large numbers of patients and repeated assessments), possibly less sensitive than a comprehensive neurocognitive assessment would be. Although we cannot rule out delayed effects on neurocognitive function or neurological events after longer periods on monotherapy than those in this study, the size and duration of our trial nevertheless provides reassurance that even if monotherapy were to have less brain penetration, this factor is unlikely to have important clinical negative effects.

The strengths of this trial are its size and duration (more than three times the randomly allocated person follow-up time of previous studies, allowing precise estimates of important but uncommon efficacy and safety outcomes); low withdrawal and loss-to-follow-up; pragmatic design set in routine clinical care, with flexibility in drug selection in both treatment groups; and use of a primary efficacy endpoint relevant to long-term management. The open-label design, a possible limitation, is unlikely to have affected detection of viral rebound or resistance (and hence the primary endpoint) because the protocol-mandated frequency of viral load testing was identical in the two groups (apart from two extra tests at weeks 4 and 8 in the monotherapy group), and we did resistance testing in everyone with viral rebound. Likewise, the open-label design is unlikely to have affected adverse event detection because the frequency of laboratory safety monitoring was standardised and adverse events were graded with standardised diagnostic criteria. Another possible limitation is the absence of before-treatment resistance tests that would help rule out resistance acquired before the trial. Although entry criteria were broad, patients entering this trial were established for years on a stable effective regimen, yet needed to be willing to change to an alternative regimen (in some cases adding new drugs with risk of side-effects or increased pill burden) if randomly allocated to do so. In view of the absence of any certainty of benefit, only a few eligible patients would therefore have been likely to be prepared to participate. Such motivated patients might have better outcomes (perhaps mediated by adherence) than the general clinic population would have, and this notion is consistent with the very low amount of rebound and resistance noted in the OT group. The main study finding that, despite high levels of viral load rebound, protease inhibitor monotherapy does not jeopardise future treatment options is likely to be generalisable internationally to settings where treatment is individualised and regular viral load monitoring done. However, protease inhibitor monotherapy is not appropriate for resource-limited settings delivering treatment without regular viral load monitoring.^36^

Protease inhibitor monotherapy is an acceptable alternative to standard combination ART for long-term HIV management, with a slight benefit in terms of a reduction of renal toxic effects. The need for regular viral load monitoring and a possible switch back to combination treatment might be perceived as drawbacks, but this approach could nevertheless appeal to patients who are stable on treatment but wish to minimise their exposure to specific drugs or more than one drug class. Broadly, the trial challenges the notion that combination treatment is essential for management of chronic HIV infection.

## Figures and Tables

**Table 1:** Baseline characteristics

		**OT (n=291)**	**PI-mono (n=296)**	**Total (n=587)**
**Demographic and clinical characteristics**				
Age (years)		43 (37–49, 23–75)	45 (39–50, 23–67)	44 (38–49, 23–75)
Route of infection				
	Homosexual	175 (60%)	176 (59%)	351 (60%)
	Heterosexual	108 (37%)	108 (36%)	216 (37%)
	Other	8 (3%)	12 (4%)	20 (3%)
Female		64 (22%)	73 (25%)	137 (23%)
Ethnic origin				
	White	206 (71%)	195 (66%)	401 (68%)
	Black	73 (25%)	90 (30%)	163 (28%)
	Other	12 (4%)	11 (4%)	23 (4%)
Hepatitis C virus antibody positive		7 (2%)	14 (5%)	21 (4%)
**HIV disease status**				
Previous AIDS-defining illness		59 (20%)	57 (19%)	116 (20%)
Nadir CD4 count (cells per μL)		181 (90–258)	170 (80–239)	178 (86–250)
Baseline CD4 count (cells per μL)		512 (386–658)	516 (402–713)	513 (392–682)
Undetectable baseline HIV viral load		276 (95%)	279 (94%)	555 (95%)
Duration of undetectable viral load (months)		36 (17–62)	38 (22–66)	37 (20–63)
**ART history**				
Years since ART started		3·9 (2·0–6·4)	4·2 (2·4–6·9)	4·0 (2·2–6·7)
On first ART combination		91 (31%)	96 (32%)	187 (32%)
Number of drugs ever received		5 (3–6)	4 (3–6)	4 (3–6)
NNRTI at entry				
	Any	157 (54%)	157 (53%)	314 (53%)
	Efavirenz	115 (40%)	115 (39%)	230 (39%)
	Nevirapine	42 (14%)	39 (13%)	81 (14%)
	Etravirine	0	3 (1%)	3 (1%)
Protease inhibitor at entry				
	Any	134 (46%)	139 (47%)	273 (47%)
	Atazanavir	59 (20%)	59 (20%)	118 (20%)
	Lopinavir	28 (10%)	49 (17%)	77 (13%)
	Darunavir	24 (8%)	13 (4%)	37 (6%)
	Saquinavir	16 (5%)	15 (5%)	31 (5%)
	Fosamprenavir	7 (2%)	3 (1%)	10 (2%)
NRTIs at entry				
	Any	291 (100%)	296 (100%)	587 (100%)
	Emtricitabine and tenofovir	190 (65%)	180 (61%)	370 (63%)
	Lamivudine and abacavir	80 (27%)	82 (28%)	162 (28%)
	Other	21 (7%)	34 (11%)	55 (9%)
**Resistance**				
Resistance test result available before trial		181 (62%)	165 (56%)	346 (59%)
Intermediate-level or high-level resistance to NRTI or NNRTI before trial*		4 (2%)	7 (4%)	11 (3%)

Data are median (IQR, range), median (IQR), or n (%). OT=ongoing triple therapy. PI-mono=protease inhibitor monotherapy. ART=antiretroviral therapy. NNRTI=non-nucleoside reverse transcriptase inhibitor. NRTI=nucleoside reverse transcriptase inhibitor.

* Percentages are of the numbers of patients with resistance test results available before the trial.

**Table 2:** Individual patients with loss of future drug options by end of trial

	**Drugs received during trial**	**Reverse transcriptase mutations**	**Protease mutations**	**Lost drug options**
**OT**				
1*	ABC, 3TC, ATV	Val118Ile, Val179Asp, Met184Val	Ile84Val	3TC, FTC, ATV, SQV, FPV, TPV
2	TDF, FTC, RPV, DRV	Leu100Ile, Lys103Asn, Met184Val	Ala71Val	3TC, FTC, NVP, EFV, ETV, RPV
3	TDF, FTC, ETV, NVP, EFV, DRV	Lys65Arg, Glu138Ala, Tyr181Cys, Met184Val/Ile, His221Tyr, Met230Leu	..	3TC, FTC, ABC, TDF, NVP, EFV, ETV, RPV
4*	TDF, FTC, DRV	Val106Ala	..	NVP,‡ EFV‡
**PI-mono**				
5*	ATV	..	Lys20Thr, Ile50Leu/Ile, Ala71Thr	ATV
6*	DRV	..	Leu90Met	SQV†
7*	DRV	..	Ala71Thr, Leu90Met	SQV†
8*	DRV	Lys103Asn	..	NVP,‡ EFV‡
9*	DRV	Lys103Asn	..	NVP,‡ EFV‡
10*	DRV	Met41Leu, Thr215Asp	..	ZDV‡

OT=ongoing triple therapy. ABC=abacavir. 3TC=lamivudine. ATV=atazanavir. FTC=emtricitabine. SQV=saquinavir. FPV=fosamprenavir. TPV=tipranavir. TDF=tenofovir. RPV=rilpivirine. NVP=nevirapine. EFV= efavirenz. ETV=etravirine. DRV=darunavir. PI-mono=protease inhibitor monotherapy. ZDV=zidovudine.

* Met primary outcome at 3 years.

† Possibly archived resistance.

‡ Probably archived resistance (excluded in sensitivity analysis).

**Table 3:** Secondary outcomes

		**OT (n=291)**	**PI-mono (n=296)**	Difference (95% CI)*	**p value**
Serious drug-related or disease-related complications					
	Total	8 (2·7%)	15 (5·1%)	2·3% (−0·8 to 5·4)	0·15
	Death†	1 (0·3%)	6 (2·0%)	1·7% (−0·3 to 3·6)	0·12
	AIDS-defining event	1 (0·3%)	1 (0·3%)	0·0% (−1·3 to 1·3)	1·00
	Serious non-AIDS event	7 (2·4%)	12 (4·1%)	1·6% (−1·2 to 4·5)	0·26
CD4 change (cells per mm^3^)		93 (10)	109 (9)	16 (−11 to 42)	0·30
Serious adverse event		45 (15·5%)	56 (18·9%)	3·5% (−2·6 to 9·6)	0·27
Total grade 3 or 4 adverse event		159 (54·6%)	137 (46·3%)	−8·4% (−16·4 to −0·3)	0·043
Clinical grade 3 or 4 adverse event		49 (16·8%)	65 (22·0%)	5·1% (−1·3 to 11·5)	0·12
EGFR					
	<60 mL/min per 1·73 m^2^‡	28/290 (9·7%)	15/296 (5·1%)	−4·6% (−8·8 to −0·4)	0·033
	Change (mL/min per 1·73 m^2^)	−5·13 (0·67)	−3·83 (0·66)	1·30% (−0·55 to 3·15)	0·092
Symptomatic peripheral neuropathy§		44/283 (15·5%)	46/289 (15·9%)	0·4% (−5·6 to 6·3)	0·90
Facial lipoatrophy¶		23/282 (8·2%)	35/289 (12·1%)	4·0% (−1·0 to 8·9)	0·12
Abdominal fat accumulation¶		47/274 (17·2%)	57/277 (20·6%)	3·4% (−3·1 to 10·0)	0·30
10 year cardiovascular disease risk change		1·32 (0·31)	1·59 (0·31)	0·27 (−0·58 to 1·12)	0·52
Neurocognitive function change (NPZ-5 score)		0·53 (0·04)	0·52 (0·04)	−0·01 (−0·11 to 0·09)	0·94
Quality of life change					
	Mental health score	−0·75 (0·57)	−1·82 (0·54)	−1·07 (−2·61 to 0·47)	0·17
	Physical health score	−0·76 (0·53)	−1·17 (0·46)	−0·41 (−1·79 to 0·98)	0·56

Data are n (%), n/N (%), or mean (SE). Means are predicted means adjusted for baseline values. The numbers of patients are the numbers of patients having the specified event during the entire trial follow-up period. OT=ongoing triple therapy. PI-mono=protease inhibitor monotherapy. EGFR=estimated glomerular filtration rate.

* Difference between PI-mono and OT. Absolute differences for binary outcomes or mean differences for continuous outcomes shown.

† Causes of death were metastatic adenocarcinoma in the OT group and suicide, pulmonary embolism, breast carcinoma (recurrent), small-cell lung carcinoma, glioblastoma, and anal carcinoma in the PI-mono group (details in appendix p 4).

‡ New episodes after baseline.

§ Symptomatic peripheral neuropathy at one or more of the post-baseline-scheduled follow-up assessments (irrespective of status at baseline).

¶ Facial lipoatrophy present at one or more of the post-baseline-scheduled follow-up assessments (irrespective of status at baseline), assessed by a doctor or nurse; abdominal fat accumulation at last available assessment compared with baseline (irrespective of status at baseline), self-assessed by the patient.
