# Supplementary material for: Protease inhibitor monotherapy for long-term management of HIV infection: a randomised, controlled, open-label, non-inferiority trial
Source: Lancet HIV. 2015 Sep 14;2(10):e417–26. doi: 10.1016/S2352-3018(15)00176-9 (PMC4765553; doi:10.1016/S2352-3018(15)00176-9)
Supplement: Supplementary appendix [file mmc1.pdf]

THE LANCET HIV

Supplementary appendix

This appendix formed part of the original submission and has been peer reviewed. We post it as supplied by the authors.

Supplement to: Paton NI, Stöhr W, Arenas-Pinto A, et al. Protease inhibitor monotherapy for long-term management of HIV infection: a randomised, controlled, open-label, non-inferiority trial. *Lancet HIV* 2015; published online Sept 15. http://dx.doi.org/10.1016/S2352-3018(15)00176-9.

SUPPLEMENTARY MATERIALS

Randomised controlled trial of a protease inhibitor monotherapy strategy for the long-term management of HIV infection

Paton NI, Stöhr W, Arenas-Pinto et al.

SUPPLEMENTARY TABLE 1: Resistance test results in cases meeting the definition of the primary endpoint, loss of future drug options*

Patient	Cumulative ART exposure before trial entry		Time of resistance test (trial week)†	Cumulative ART exposure during trial ‡		Viral load (c/ml) at time of resistance test	Drug-resistance mutations present§		Drugs with reduced susceptibility¶		
	NRTI, NNRTI	PI		NRTI, NNRTI,II	PI		RT	PRO	NRTI	NNRTI	PI
**OT group**											
1	ZDV, 3TC, NVP, ABC	ATV	-89	-	-	-	118I, 179D		-	-	-
			70	ABC, 3TC	ATV	33,300	118I, 179D, 184V	84V	3TC^{H}, FTC^{H}	-	SQV^{H}, TPV^{I}, ATV^{I}, FPV^{H}
			185	TDF, FTC, RAL (3TC, ABC)	DRV (ATV)	4,100	118I, 179D	-	-	-	-
2	d4T, TDF, EFV, FTC	DRV	-286	-	-	-	-	71V	-	-	-
			193	TDF, FTC, RPV	(DRV)	1,500	100I, 103N, 184V	71V	3TC^{H}, FTC^{H}	NVP^{H}, EFV^{H}, ETV^{I}, RPV^{I}	-
3	DDI, 3TC, EFV, FTC, TDF	-	161	TDF, FTC, ETV (EFV, NVP)	(DRV)	60	138A, 184V/I	-	3TC^{H}, FTC^{H}		-
			167	TDF, FTC, ETV (EFV, NVP)	(DRV)	5,400	65R, 138A, 181C, 184V/I, 221Y, 230L	-	3TC^{H}, FTC^{H}, ABC^{H}, TDF^{I}	NVP^{H}, EFV^{H}, ETV^{H}, RPV^{H}	-
4	3TC, ABC	SQV, LPV	-179				-	-	-	-	-
			51	TDF, FTC	DRV	2,300	106A	-	-	NVP^{H}, EFV^{I}	-
**PI-mono group**											
5.	FTC, TDF, EFV	-	-165	-	-	-	-	71T	-	-	-
			48	-	ATV	23,400	-	20T, 50L/I,71T	-	-	ATV^{H}
			57	-	ATV	3,300	-	20T, 71T	-	-	-
			61	-	ATV	2,400	-	71T	-	-	-

6.	ZDV, 3TC, ABC, EFV	LPV	155	-	DRV	200	-	90M	-	-	SQV^{I}
7.	FTC, TDF, EFV	-	73	-	DRV	300	-	71T, 90M	-	-	SQV^{I}
8.	3TC, ABC, EFV	-	82	-	DRV	<20	103N	-	-	NVP^{H}, EFV^{H}	-
9.	ZDV, 3TC, FTC, TDF, NVP	SQV	-290	-	-	-	-	11I	-	-	-
			83	-	DRV	1,100	103N	-		NVP^{H}, EFV^{H}	-
10.	ZDV,3TC	LPV	17	-	DRV	300	41L,215D	-	ZDV^{I}	-	-

* Drugs used for less than 28 days are not reported. ART, antiretroviral therapy; ABC, abacavir; d4T, stavudine; 3TC, lamivudine; FTC, emtricitabine; TDF, tenofovir; ZDV, zidovudine; SQV, saquinavir; FPV fosamprenavir; TPV tipranavir; ATV, atazanavir; NVP nevirapine; EFV , efavirenz; ETV etravirine; RPV, rilpivirine; RAL, raltegravir

† A negative value refers to the timing of a pre-trial resistance test relative to randomisation (usually obtained before start of antiretroviral therapy) if performed.

‡ Shown is the cumulative drug exposure between randomisation and the time of performing the resistance test. PI-mono patients continued NRTIs for the first 14 days after randomisation (drugs not shown). Drugs taken during the trial but discontinued prior to the resistance test are shown in parentheses.

§ Drug resistance mutations limited to those used in the Stanford algorithm.

¶ Drug susceptibility determined from Stanford algorithm. List limited to drugs that are included as treatment options in British HIV Association 2012 treatment guidelines, with the addition of saquinavir. Superscript denotes level of predicted resistance: I=intermediate, H = high

Supplementary Table 2: Causes of death, risk factors and HIV disease status during trial follow-up prior to the presentation of the terminal condition

Patient no. (group)	Cause of death (week of presentation of terminal condition)	Clinical history and risk factors	HIV disease status from trial entry to presentation of terminal condition
1 (OT)	Metastatic adenocarcinoma, probable lung origin (week 20)	58y male; 40 pack-year history of smoking; presented with right thigh mass; mediastinal and adrenal mass on CT	CD4 525 at baseline; VL suppressed from randomisation to presentation of terminal condition
2. (PI-mono)	Trauma, presumed suicide (week 17)	47y male; no history of depression	CD4 215 at baseline; VL suppressed from randomisation to death
3. (PI-mono)	Pulmonary embolism (week 51)	40y female; non-smoker; hospitalisation for encephalitis (week 40 to 50); pulmonary embolism secondary to deep venous thrombosis	CD4 333 at baseline; VL rebound week 36 due to non-adherence; re-suppressed partially with re-introduction of combination therapy
4. (PI-mono)	Breast carcinoma, recurrent (week 7)	54y female; non-smoker; angiosarcoma of breast 2y before study entry treated by mastectomy;	CD4 550 at baseline; VL supressed from randomisation to presentation of terminal condition
5. (PI-mono)	Small-cell lung carcinoma (week 178)	59y male: smoker for 30 years; presented with headache; CT lung mass, biopsy showed small cell carcinoma of lung	CD4 208 at baseline: VL suppressed from randomisation to presentation of terminal condition
6. (PI-mono)	Glioblastoma (week 66)	61y male; non-smoker; presented with headache at week 66; mass on CT head, biopsy showed high grade glioblastoma	CD4 468 at baseline; VL rebound weeks 25-32 (< 300 c/ml). Re-suppressed with addition of TDF/FTC thereafter.
7. (PI-mono)	Anal carcinoma (week 80)	56y male; smoker; anal mass detected; biopsy showed squamous cell carcinoma	CD4 319 at baseline; VL rebound weeks 24-43 (max 815 c/ml). Re-suppressed with addition of TDF/FTC thereafter.

Supplementary Table 3: Serious Adverse Events by category of event*

	**OT**	**PI-mono**
Total events	61	75
Death	1	6
Life threatening	4	2
Caused / prolonged hospitalisation	58	67
Disability / incapacity	0	2
Congenital anomaly / birth defect	0	0
Other	4	5

*Shown are the number of events in each category

Supplementary Table 4: Grade 3 or 4 adverse events by Clinical and laboratory category *

	OT	PI-mono	Difference (95% CI) †	P
**Clinical events**				
Total	49 (17%) [78]	65 (22%) [91]	5.1% (-1.3% to 11.5%)	0.12
Cardiovascular	8 (3%) [11]	7 (2%) [7]	-0.4% (-2.9% to 2.2%)	0.77
Respiratory	5 (2%) [5]	11 (4%) [11]	2.0% (-0.6% to 4.6%)	0.14
Gastrointestinal	12 (4%) [15]	7 (2%) [8]	-1.8% (-4.6% to 1.1%)	0.23
Hepatic	2 (1%) [2]	3 (1%) [3]	0.3% (-1.4% to 2.1%)	1.0
Renal‡	2 (1%) [2]	3 (1%) [3]	0.3% (-1.4% to 2.1%)	1.0
CNS§	9 (3%)[10]	17 (6%) [20]	2.7% (-0.7% to 6.0%)	0.12
Skin	7 (2%) [7]	9 (3%) [9]	0.6% (-2.0% to 3.3%)	0.64
Other	20 (7%) [26]	24 (8%) [30]	1.2% (-3.0% to 5.5%)	0.57
**Laboratory events**				
Total	131 (45%) [158]	97 (33%) [117]	-12.2% (-20.1% to -4.4%)	0.0023
Phosphate decreased	73 (25%)	37 (13%)	-12.6% (-18.8% to -6.3%)	<0.0001
Bilirubin increased	44 (15%)	21 (7%)	-8.0% (-13.1% to -3.0%)	0.0019
Lipids increased	22 (8%)	39 (13%)	5.6% (0.7% to 10.5%)	0.026
Haematological	8 (3%)	5 (2%)	-1.1% (-3.4% to 1.3%)	0.38
Other	7 (2%)	11 (4%)	1.3% (-1.5% to 4.1%)	0.36

*Shown are the numbers of patients and the percentage of patients with a given type of Grade 3 or 4 adverse events, and the total number of events in each category. Recurrent laboratory events in the same patient were counted as a single event. P values were calculated for chi-square or Fisher's exact tests for the proportion of patients affected.

† Shown are absolute differences in the proportions of patients affected in each group

‡ Renal events were nephrolithiasis, pyelonephritis in OT group and renal cell carcinoma, end-stage renal failure (progression of existing chronic renal impairment present at study entry) and acute renal impairment (transient, accompanying episode of pneumonia) in PI-mono group.

§ CNS events were depression and/or suicidal ideation (4), anxiety/stress (2), headache (2), psychosis (1), and normal pressure hydrocephalus (1) in the OT group; depression and/or suicidal ideation (9), anxiety/stress (1), headache (2), myasthenia gravis (1), psychosis (2); psychiatric symptoms, unspecified (1), meningioma (1), glioblastoma (1), head injury (1) and convulsion (1) in the PI-mono group

Supplementary Table 5: Adverse events that were considered as related to antiretroviral therapy *

	OT	PI-mono	Difference (95% CI)	P
Total	6 (2.1%) [6]	3 (1.0%) [3]	-1.0% (-3.2% to 1.1%)	0.34
Death	0	0		
SAE	2	0		
Grade 3/4	3	3		
SAE + Grade 3/4	1	0		

* Shown are the total numbers of patients and the percentage of total patients with any event considered as possibly, probably or definitely related to antiretroviral therapy based on independent review at the coordinating centre. The number of events per category are shown for the individual categories. P value was calculated using Fisher's exact test for the proportion of patients affected.

SUPPLEMENTARY FIGURE 1: Outcome of confirmed viral load rebound episodes in PI-mono arm

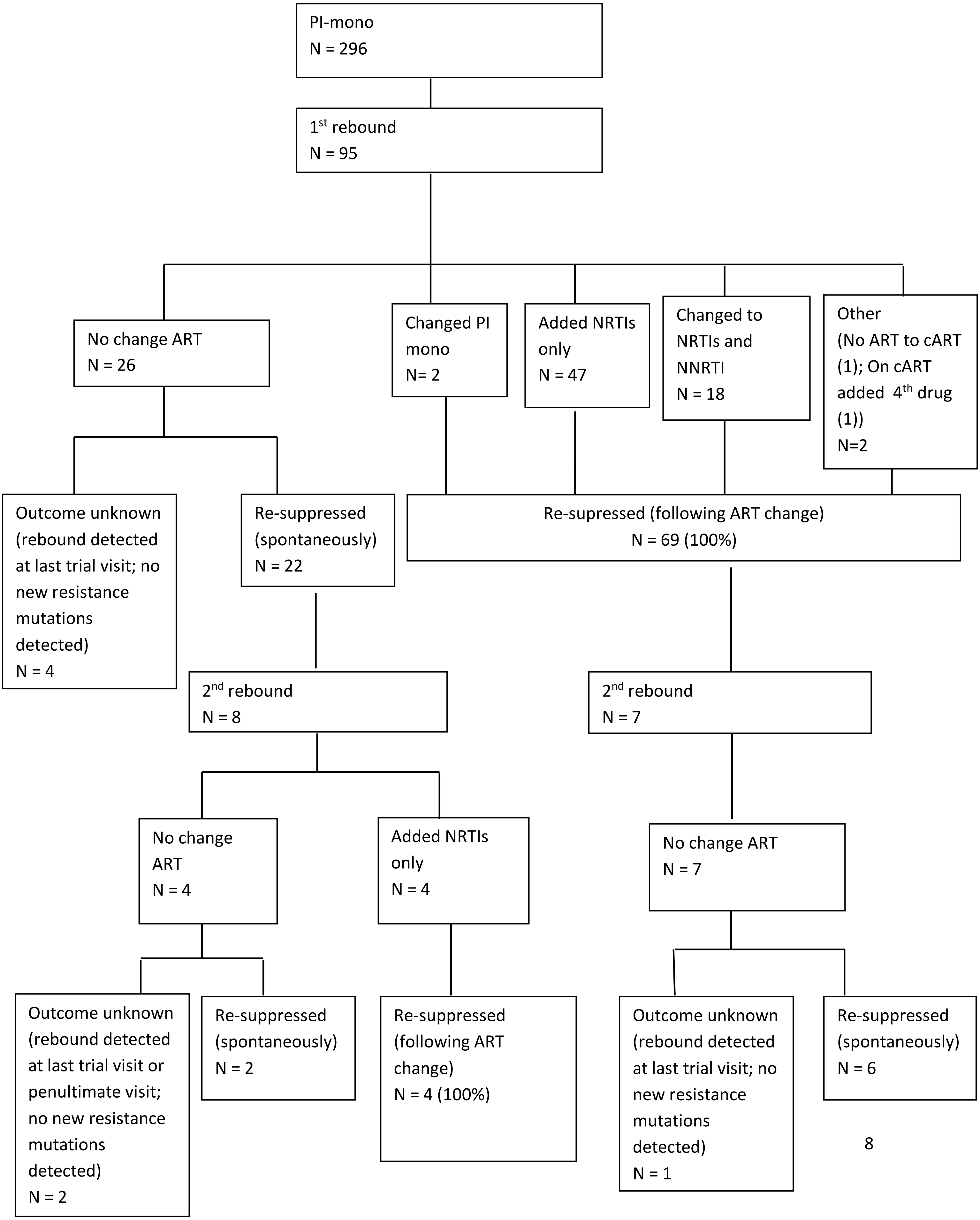

SUPPLEMENTARY FIGURE 2: Time to virological rebound - extended definition

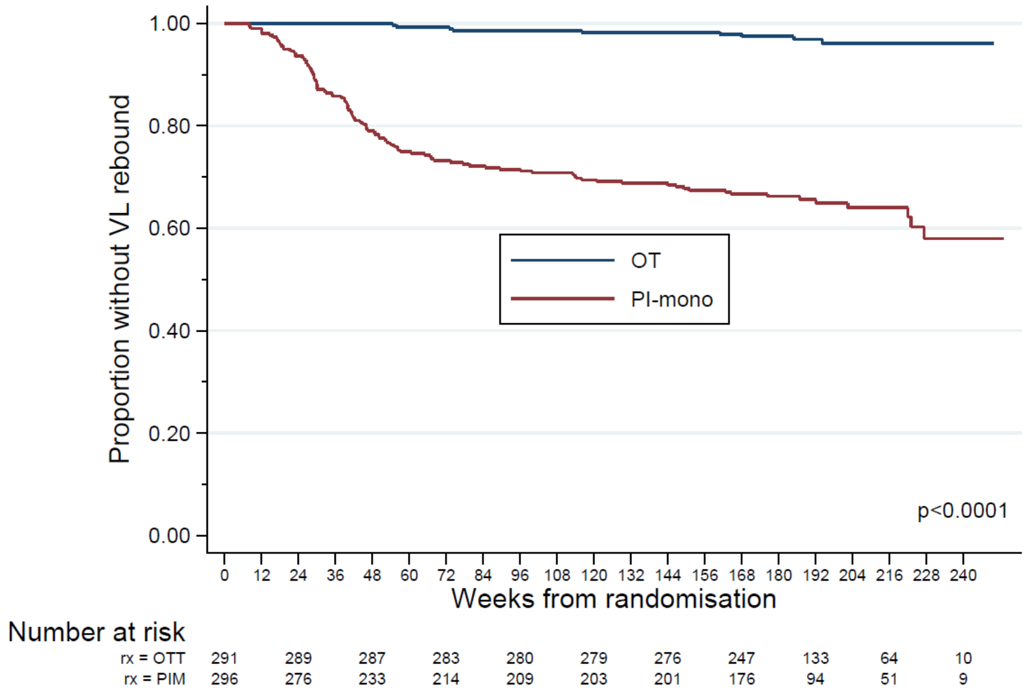

Extended definition of VL rebound included protocol-defined confirmed VL rebound cases as well as those that were changed earlier (i.e. at less than 4 weeks after the first detectable VL or after only 2 detectable VLs). Number (proportion) of rebound by end of follow-up: 9 (4.0%) in OT, and 105 (42.0%) in PI-mono (risk difference 38.0% [95%-CI: 29.2-46.8%]; P < 0.001). In PI-mono, rate of rebound is 27 per 100 person years in the first year, and 6 per 100 person years in subsequent years.
